# Dental management of patients receiving anticoagulant and/or antiplatelet treatment

**DOI:** 10.4317/jced.51215

**Published:** 2014-04-01

**Authors:** Ana Mingarro-de-León, Begonya Chaveli-López, Carmen Gavaldá-Esteve

**Affiliations:** 1 Degree in Dental Surgery. Master in Oral Medicine and Surgery. Valencia University Medical and Dental School. Valencia, Spain; 2 Associate Professor of Oral Medicine. Valencia University Medical and Dental School. Valencia, Spain

## Abstract

Introduction: Adequate hemostasis is crucial for the success of invasive dental treatment, since bleeding problems can give rise to complications associated with important morbidity-mortality. The dental treatment of patients who tend to an increased risk of bleeding due to the use of anticoagulant and/or antiplatelet drugs raises a challenge in the daily practice of dental professionals. Adequate knowledge of the mechanisms underlying hemostasis, and the optimized management of such patients, are therefore very important issues. Objectives: A study is made of the anticoagulant / antiplatelet drugs currently available on the market, with evaluation of the risks and benefits of suspending such drugs prior to invasive dental treatment. In addition, a review is made of the current management protocols used in these patients. Material and Methods: A literature search was made in the PubMed, Cochrane Library and Scopus databases, covering all studies published in the last 5 years in English and Spanish. Studies conducted in humans and with scientific evidence levels 1 and 2 (metaanalyses, systematic reviews, randomized phase 1 and 2 trials, cohort studies and case-control studies) were considered. The keywords used for the search were: tooth extraction, oral surgery, hemostasis, platelet aggregation inhibitors, antiplatelet drugs, anticoagulants, warfarin, acenocoumarol. Results and Conclusions: Many management protocols have been developed, though in all cases a full clinical history is required, together with complementary hemostatic tests to minimize any risks derived from dental treatment. Many authors consider that patient medication indicated for the treatment of background disease should not be altered or suspended unless so indicated by the prescribing physician. Local hemostatic measures have been shown to suffice for controlling possible bleeding problems resulting from dental treatment.

** Key words:**Tooth extraction, oral surgery, hemostasis, platelet aggregation inhibitors, antiplatelet drugs, anticoagulants, warfarin, acenocoumarol.

## Introduction

Hemostasis is a defense mechanism composed of a series of independent biological systems that aim to preserve vascular integrity and avoid blood losses, while ensuring optimum fluidity throughout the circulatory system (1,2).

Hemostatic alterations have a broad range of potential causes, including deficiency states, hereditary and metabolic alterations, cancer, etc. However, at present, the most frequent cause of blood coagulation disorders in developed countries is the use of drug substances (3). Many drug products are available for the prevention of thromboembolic events; it is therefore very important for dental professionals to know these products, their mechanisms of action, and the measures of caution required in order to prevent complications.

**Figure 1:**
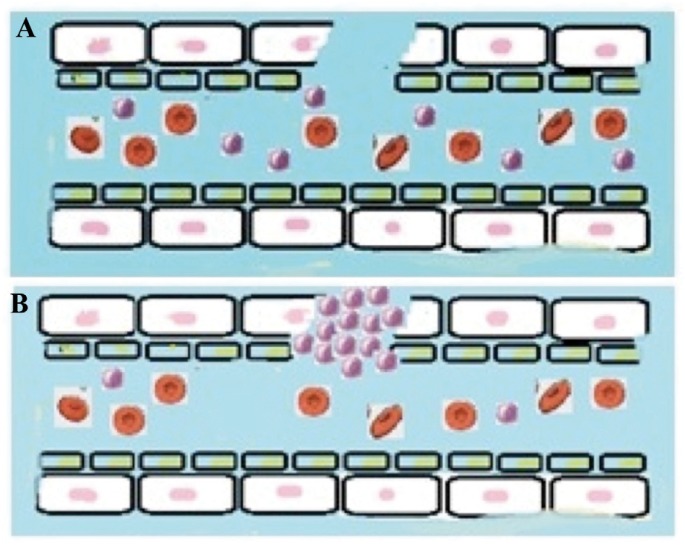
Phases of hemostasis: A) Vascular or vasoconstriction phase. B) Platelet phase.

Tissue damage is generally associated to vascular injury resulting in more or less profuse bleeding (2). Vascular endothelial rupture exposes different proteins of the subendothelial tissue layer to the bloodstream, triggering three different hemostatic mechanisms (2-5):

- Vascular or vasoconstriction phase: Vasoconstriction of the damaged blood vessel occurs immediately afte vascular injury, mediated by the vascular smooth muscle (Fig. 1), and reduces blood loss from the damaged vessel. Such vasoconstriction lasts about 20 minutes. The vasoconstrictive response alone is not sufficient to stop bleeding, but it does have two important effects: it reduces blood loss and triggers the second phase, facilitating platelet adhesion secondary to exposure of the subendothelial collagen fibers and basal membrane of the damaged blood vessel wall (2,5).

- Platelet phase, or platelet clot formation: The purpose of this phase is to form a primary hemostatic clot composed of aggregated platelets (2) (Fig. 1). Platelets are small cell fragments derived from the megakaryocytes of the bone marrow, and have a half-life of 7-10 days. Their main function is to maintain vascular integrity and form a platelet clot in the event of vascular damage (4). The normal count is between 150,000 and 400,000 platelets/mm3, and the platelet maturation sequence lasts 3-5 days (4). The platelets adhere to the subendothelial collagen exposed as a result of vascular damage, and aggregate to form a platelet clot (4) that contributes to reduce blood loss (i.e., bleeding).

- Plasmatic phase, or production of fibrin that stabilizes and reinforces the platelet clot (coagulation): This phase is characterized by a complex series of proteolytic reactions known as the coagulation cascade. The classical cascade comprises two pathways: extrinsic and intrinsic, which in turn merge to form a common pathway (1).

The plasmatic coagulation phase involves the transformation of fibrinogen (soluble) into fibrin (insoluble), mediated by thrombin – a proteolytic enzyme formed by the activation of prothrombin, following sequential activation of the coagulation factors (1).

The intrinsic pathway is started by the activation of factor XII through contact with the subendothelial tissues in the damaged zone. The extrinsic pathway in turn is started when blood comes into contact with the tissue thromboplastin released by the damaged tissues, with activation of factor VII. From this point a cascade of metabolic reactions involving different coagulation factors is generated, ultimately giving rise to the formation of thrombin which, as has been commented above, transforms fibrinogen into fibrin (1,2).

Finally, the blood clot is dissolved in the fibrinolytic phase. When the damaged vascular wall is repaired, activated factor XII facilitates the conversion of an inactive plasma molecule to its active form, called kallikrein. The latter in turn catalyzes the conversion of inactive plasminogen to the active molecule plasmin - an enzyme that digests fibrin, yielding degradation products and promoting dissolution of the clot. Once fibrin degradation has been completed, plasmin is quickly neutralized by antiplasmin (2,4,5).

Antiplatelet or platelet aggregation inhibitor drugs inhibit the aggregation of platelets, thereby avoiding platelet clot formation and suppressing the first hemostatic phase (4). In contrast, anticoagulant drugs inhibit the enzyme vitamin K reductase, which mediates conversion of vitamin K epoxide to its active form. As a result, the formation of coagulation factors dependent upon this active form is inhibited, and the coagulation process is blocked (6). At clinical level, the end result following surgery is the same in both cases, i.e., hemostatic alteration that may result in bleeding which can prove difficult to control. As a result, some authors (7,8) recommend suspending, reducing or replacing antiplatelet / anticoagulation medication prior to invasive dental treatments, while others (9-10) advise against such measures, due to the possibility of an increased risk of thromboembolic events.

The present study offers an update on the different types of antiplatelet drugs and anticoagulants currently available on the market, with an evaluation of the risks and benefits of suspending such drugs prior to invasive dental treatment. In addition, a review is made of the current management protocols used in these patients.

## Material and Methods

A literature search was made in the PubMed, Cochrane Library and Scopus databases, using the key words: tooth extraction, oral surgery, hemostasis, platelet aggregation inhibitors, antiplatelet drugs, anticoagulants, warfarin, acenocoumarol, validated by the Mesh and DeCS dictionaries, and using the boolean operator “AND” for relating the terms in each search.

The search covered all studies published in dental journals with a relevant impact factor over the last 5 years in English and Spanish. Studies conducted in humans and with scientific evidence levels 1 and 2 (metaanalyses, systematic reviews, randomized phase 1 and 2 trials, cohort studies and case-control studies) were considered.

## Results and Discussion

An examination of the literature on the subject over the last few years reveals a lack of consensus on the way to deal with dental patients receiving antiplatelet medication / anticoagulants.

It remains a recommendation among some authors (7,8) to reduce or suspend antithrombotic medication two or three days before any dental treatment involving possible bleeding.

However, in recent years a growing number of investigators have adopted a more conservative approach, preferring not to interfere with drug treatment (i.e., without suspending the medication several days before dental treatment or modifying the dosing scheme), and controlling bleeding after the dental procedure by means of local hemostatic measures (9-14).

- Anticoagulant drugs and management of anticoagulated patients

Despite their well established efficacy after more than 50 years on the market, these drugs pose problems in the form of adverse effects and interactions with certain drug substances and foods. Moreover, although the antithrombotic effect begins 48-72 hours after administration, reduction of the coagulation factors does not occur until 5 days after the start of treatment (6). Consequently, the clinical use of these drugs is complicated by the need to closely monitor their activity. In effect, anticoagulants require correct monitorization and dose adjustment to obtain the desired therapeutic effect while minimizing the risk of adverse effects associated with both excess anticoagulation (bleeding) and insufficient anticoagulation (thrombosis)(6).

1. Coumarin derivatives

• Acenocoumarol or nicumalone (Sintrom® 1 and 4 mg): The half-life of this drug is quite short (8-10 hours). It is prescribed as a single daily dose, and is the most widely used drug in Spain (6).

• Warfarin sodium (Aldocumar® 10 mg, Coumadin®): This is the most widely prescribed oral anticoagulant in the United States and the United Kingdom (6). It offers prolonged action, with a half-life of 48-72 hours (6,15,16).

• Ethyl biscumacetate: This drug is not commonly used, due to its short lasting action.

• Phenprocoumon: This drug is longer acting than acenocoumarol.

• Fluindione: Little used, and only in France.

2. Indandione derivatives

These are very toxic synthetic drugs, and are therefore not used, at least in Spain. They have been associated particularly to hypersensitivity reactions (6).

3. Recent introduction on the market

• Dabigatran etexilate (Pradaxa®): This recently marketed drug is a potent inhibitor of free thrombin, thrombin bound to fibrin, and platelet aggregation induced by thrombin – thereby preventing thrombus formation. Its main indication is in elective total hip or knee replacement surgery. It is also indicated for the prevention of stroke and systemic embolism in adults with non-valvular atrial fibrillation (17-19). It requires no monitorization (17-20). The drug is administered via the oral route in the form of two daily doses of 110 mg. Therapy is started 1-4 hours after surgery and is maintained for up to 10 days day after the operation. Plasma peak concentrations are reached between 30 minutes and two hours after administration. The bioavailability is 5-6%, and the half-life after single and multiple dosing is 8 and 17 hours, respectively. (17). Most of the drug (80%) is excreted in urine (20).

• Rivaroxaban and apixaban (Xarelto®, Eliquis®): These are selective factor Xa inhibitors administered via the oral route and with an absorption of close to 100%. The available clinical data are still limited, and the existing information on their metabolism and possible drug interactions comes mainly from nonclinical studies. In the same way as dabigatran, these drugs do not require routine monitorization (17,20).

Patients treated with oral anti-vitamin K anticoagulants require periodic monitoring, based on the prothrombin time (PT). Since this parameter is somewhat imprecise, use of the INR (international normalized ratio: proportion between patient PT and control PT, standardized and corrected) is currently advised (1,2,5,6,10-16). The recommended anticoagulation levels vary between INR 2-3 for all indications, with the exception of patients with heart valve implants, in which INR should be maintained between 2.5-3.5 (2,6).

The literature review identified articles comparing a control group with anticoagulant suspension versus an experimental group in which anticoagulation is maintained. As an example, Evans *et al*. (21) compared 109 patients divided into two groups: warfarin was suspended two days before the operation in one group, and was continued in the other group with the maintenance of INR 2-4. Mild postoperative bleeding was recorded in 15 patients who continued warfarin and in 7 patients in which warfarin was suspended – the difference being nonsignificant. Bleeding was controlled by applying pressure with a piece of gauze or dressing in the bleeding zone.

Méndez *et al*. (22) adopted a similar procedure but with a much smaller study sample. In this case there were 15 patients in the group in which anticoagulation was maintained, and 10 patients in the group in which such treatment was suspended two days before the operation. Minimum postoperative bleeding was recorded in four of the patients in which anticoagulation was maintained, and in none of the controls. However, the small sample size did not allow the recording of statistically significant results.

Al-Mubarack *et al*. (23) in turn compared 214 patients distributed into four groups, establishing a combination between warfarin suspension and continuation, with or without suture application. These authors observed slightly greater bleeding immediately after tooth extraction in the two groups in which warfarin was maintained. The number of cases was not specified, though the differences were described as nonsignificant.

Sacco *et al*. (10) compared an experimental group in which anticoagulation was not suspended versus a control group in which dose reduction was indicated two days before the operation. These authors recorded no cases of immediate bleeding, though bleeding was observed two hours after the operation, and proved slightly greater in the experimental group.

Bajkin *et al*. (11) likewise found no significant differences on comparing two groups of approximately 110 patients each (anticoagulation being suspended three days before surgery in one group and maintained in the other).

As indicated by these results, no significant differences are observed in the prevalence of bleeding in one group versus the other – thus supporting the opinion of the above authors that it is not necessary to either suspend or reduce anticoagulation in the context of minor surgery, since local hemostatic measures suffice to minimize bleeding.

**Figure 2:**
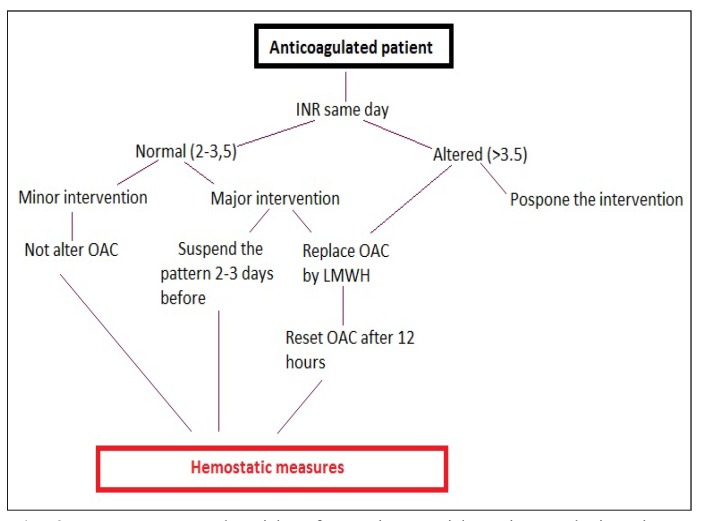
Management algorithm for patients with anticoagulation therapy scheduled for invasive dental treatment. OAC: oral anticoagulant; LMWH: low molecular weight heparin.

On the other hand, in the case of elective surgery, major surgery (over three extractions, implant placement, etc.), INR > 3.5 and other concomitant risk factors, the protocol of choice is to suspend anticoagulation 2-3 days before surgery (depending on the risk of thromboembolism), or to switch to subcutaneous heparin (6) (Fig. 2).

Regarding the new oral anticoagulant drugs, the data in relation to dental treatment are still limited, since these drugs have only recently appeared on the market. In principle, it seems that there is no need to suspend anticoagulation or modify the dose in the case of operations with a normal or low bleeding risk (simple extractions, operations lasting under 45 minutes)(17,19,24).

In the case of major surgery or procedures involving a high bleeding risk (multiple extractions, operations lasting > 45 minutes, head and neck cancer surgery) (24), the recommendation is to suspend the medication 24 hours before the operation and to reintroduce it after 24 hours, provided good hemostasis has been achieved (17,19). In contrast, Spyropoulus *et al*. (24) in these cases advises anticoagulant suspension 2-3 days before the operation – though the authors underscore the need to carry out more studies and to investigate the possible effects of these new drugs.

Apart from their rapid action and few interactions, one of the main advantages of the new anticoagulants seems to be that they require no monitorization (17,19,24,25). Nevertheless, there are situations in which we need to know whether the level of anticoagulation is correct, e.g., in emergency surgery, to assess treatment compliance, or even to reassure the patient that the anticoagulation levels are correct (25). In these cases both activated partial thromboplastin time (aPTT) and thrombin time (TT) may prove useful (17,25).

- Antiplatelet drugs and management of patients with antiplatelet medication

Antiplatelet drugs are generally prescribed for the prevention of arterial and venous thrombosis in patients with ischemic heart disease, heart valve implants and stents, and in people at risk of suffering cerebrovascular events such as stroke. Since these drugs act by inhibiting platelet function, they have been accepted as adequate antithrombotic treatment (26). The main antiplatelet drugs marked in Spain are the following:

• Acetylsalicylic acid (ASA)(Aspirin®, Adiro®, Biopak®, Tromalyt®) blocks thromboxane A2 production, thereby inhibiting cyclooxygenase activity and consequently platelet aggregation (4). The effect of this drug upon the platelets is irreversible, and therefore lasts for the full length of platelet life (7-10 days). Low doses (75-100 mg) are generally indicated in cases of chest pain, ischemia, transient ischemic accidents, and during the postoperative period (post-angioplasty / angiography) (27).

• Clopidogrel bisulfate (Plavix®, Iscover®) inhibits platelet aggregation by blocking ADP binding to its platelet receptor and subsequent activation of the GPIIb-IIIa complex mediated by ADP (4,27).

 • Ticlopidine hydrochloride (Tiklid®, Ticlodone®) inhibits platelet binding to ADP-fibrinogen, along with posterior platelet-platelet binding (aggregation) (4).

• Dipyridamole (Persantin®) blocks adenosine transport in platelets, erythrocytes and endothelial cells. The resulting increase in the local extracellular concentrations of adenosine acts directly upon the platelet A2-receptors, increasing the platelet cyclic adenosine monophosphate (cAMP) levels and consequently blocking platelet aggregation (4,27).

• Triflusal (Disgren®) is an acetylsalicylic acid (aspirin) analog that blocks platelet aggregation by irreversibly inhibiting platelet cyclooxygenase (26).

Because of their potential bleeding effect, antiplatelet drugs are often interrupted during the perioperative period, without adequately evaluating the increased thrombotic risk of this decision. More recent publications suggest that the increase in bleeding risk induced by antiplatelet drugs has been exaggerated, while at the same time the increased thrombotic risk associated with treatment interruption has been underestimated (28). Consequently, although each invasive dental procedure implies a risk of oral bleeding, it is not advisable to interrupt antiplatelet therapy, since the increased risk of thromboembolism could outweigh the risk of bleeding (26-28).

Madan *et al*. (27) studied 51 patients subjected to antiplatelet treatment with low-dose ASA (75-100 mg). The medication was not suspended in any of the patients, and in no case was bleeding observed. As hemostatic measures, the authors used surgical gauze impregnated with a feracrylum solution, and 3/0 sutures.

In the same line of research, Aframian *et al*. (28) concluded that patients administered low-dose ASA can undergo any type of oral treatment, minor surgery and/or tooth extractions, without having to suspend the antiplatelet medication, and without this decision implying any added risk for the patients – local hemostatic measures being more than enough to secure bleeding control (27,28).

In 2008, Krishnan *et al*. (29) conducted a study of 82 patients divided into three groups. Antiplatelet treatment was suspended in 35 of the patients 10 days before surgery. The authors observed no significant differences among the groups, since none of them showed prolonged or significant bleeding in the operated zone.

Similar results have been reported by Morimoto *et al*. (13) in their study of 87 patients in which no increase in bleeding rate was observed despite continuation of their regular drug treatment.

Our literature review identified few studies of patients receiving two antiplatelet drugs simultaneously. Some authors have reported no increased bleeding tendency in such patients compared with individuals receiving a single antiplatelet drug (3,4). In contrast, Scharf (30) reported that dual antiplatelet treatment is associated with a 40-50% increase in bleeding risk compared with single drug treatments.

Lillis *et al*. (26), in a study of 33 patients receiving two antiplatelet drugs and 78 patients with antiplatelet monotherapy, recorded 22 cases of bleeding in the former group versus only two cases in the single drug treatment group. Although the difference was significant, the authors concluded that all of the cases of bleeding were easily controlled by local measures.

Among patients receiving dual antiplatelet treatment, special attention should focus on those carrying both conventional and drug-eluting stents (27,29,30). Although the predominant recommendation in patients receiving dual therapy is to suspend one of the two antiplatelet drugs (generally clopidogrel) between 3-5 days before the operation (31), with reintroduction 24 hours after surgery, we should avoid early discontinuation of one of the two drugs at least during the first year in such individuals. In these cases it is essential to consult the professional who prescribed the medication (30,31).

On the other hand, the results published by Lillis *et al*. (26) support the safety of dental extractions without interruptions in either simple or dual antiplatelet treatment, with the adoption of adequate local hemostatic measures.

- Postoperative recommendations and local hemostatic agents

Scully and Wolff (32), in coincidence with many other authors (6,10,13,14), recommend performing all dental surgeries in the morning, in order to be able to resolve any bleeding complications in the course of the day.

The following patient instructions are advised (11,31-33):

• Apply pressure with a piece of gauze for 30-40 minutes

• Avoid oral rinses during the first 24 hours

• Follow a soft and cold diet during the first 24 hours

• Avoid suctioning movements

• Avoid touching the socket region with the tongue or manipulation of the operated zone

The adoption of adequate hemostatic measures is the key to not having to modify the antiplatelet or anticoagulation treatment in most cases. The incidence of postoperative bleeding episodes that cannot be controlled by such measures varies between 0-3.5% (33).

As hemostatic measures, some authors recommend tranexamic acid (Amchafibrin®) as a postoperative rinse to stabilize the blood clot, since it inhibits plasminogen activation and fibrinolysis (21,22,28,31) and appears to have few side effects (nausea, diarrhea, orthostatic hypotension)(22). Rinses are advised twice a day during the first 48 hours – affording adequate hemostasis after minor surgery (28). In contrast, other authors such as Salam *et al*. (34) and Ferrieri *et al*. (9) prefer to use sterile gauze impregnated tranexamic acid rather than tranexamic acid rinses, arguing that the risk of clot dissolution as a result of the mechanical rinsing action outweighs the benefits of the antifibrinolytic agent. On the other hand, it must be mentioned that in some countries tranexamic acid either has not been approved for local hemostasis (as in Japan)(13) or is not fully accessible (as in the United Kingdom). This has favored the use of other alternative hemostatic measures (21).

Other widely used hemostatic agents are intraalveolar oxidized cellulose (Surgicel®), reabsorbable collagen sponges (Octocolagen®, Gelatamp® from Roeko), fibrin adhesive (Tissucol®) or tissue adhesives (Tisuacryl® from Dentsplay) and, of course, sutures.

Bajkin *et al*. (11) and Bacci *et al*. (12) compared groups of patients with different hemostatic measures with the purpose of assessing their effectiveness against bleeding. None of the authors found any given measure to be superior to the rest (11-13).

In relation to suturing, there are differing opinions regarding the ideal type of suture for minimizing bleeding. Some authors advocate reabsorbable sutures since they do not have to be removed – thereby avoiding trauma and minimizing bleeding risk (2,12,28). In contrast, other investigators prefer non-reabsorbable silk sutures, since they retain much less plaque and therefore greatly lessen the risk of bacterial penetration into the bloodstream (23). This in turn reduces the risk of postoperative complications such as thromboembolic phenomena or infections (13-15).

## Conclusions

In cases of invasive dental treatment (extractions or minor surgery), patient medication indicated for the treatment of background disease should not be altered or suspended unless so indicated by the prescribing physician. Local hemostatic measures are shown to suffice to control possible bleeding secondary to dental treatments.

Although bleeding complications are a source of concern and discomfort, they do not involve the same risks for patients as thromboembolic complications.

We have found no standardized protocol defining optimum management of these patients, though most authors coincide that patient health and safety must be the priority concern in all cases. In this context it is necessary to carefully evaluate the bleeding risk of the planned treatment, as well as the thrombotic risk of suppressing the anticoagulant or antiplatelet medication, on an individualized basis for each patient, with a view to providing optimum and personalized care.

## References

[1] Romney G, Glick M (2009). An updated concept of coagulation with clinical implications. J Am Dent Assoc.

[2] Jover-Cervero A, Poveda-Roda R, Bagan JV, Jimenez-Soriano Y (2007). Dental treatment of patients with coagulation factor alterations: An update. Med Oral Patol Oral Cir Bucal.

[3] Quintero Parada E, Sabater Recolons MM, Chimenos Klistner E, López López J (2004). Hemostasia y tratamiento odontológico. Av. Odontoestomatol.

[4] Partridge CG, Campbell JH, Alvarado F (2008). The effect of platelet-altering medications on bleeding from minor oral surgery procedures. J Oral Maxillofac Surg.

[5] Cañigral A, Silvestre FJ, Cañigral G, Alós M, Garcia-Herraiz A, Plaza A (2010). Evaluation of bleeding risk and measurement methods in dental patients. Med Oral Patol Oral Cir Bucal.

[6] Jiménez Y, Poveda R, Gavaldá C, Margaix M, Sarrión G (2008). An update on the management of anticoagulated patients programmed for dental extractions and surgery. Med Oral Patol Oral Cir Bucal.

[7] Devani P, Lavery KM, Howell CJ (1998). Dental extractions in patients on warfarin: is alteration of anticoagulant regime necessary?. Br J Oral Maxillofac Surg.

[8] Russo G, Corso LD, Biasnolo A, Berengo M, Pengo V (2000). Simple and safe method to prepare patients with prosthetic heart valves for surgical dental procedures. Clin Appl Thromb Hemost.

[9] Ferrieri GB, Castiglioni S, Carmagnola D, Cargnel M, Strohmenger L, Abati S (2007). Oral Surgery in Patients on Anticoagulant Treatment Without Therapy Interruption. J Oral Maxillofac Surg.

[10] Sacco R, Sacco M, Carpenedo M, Mannucci PM (2007). Oral surgery in patients on oral anticoagulant therapy: a randomized comparison of different intensity targets. Oral Surg Oral Med Oral Pathol Oral Radiol Endod.

[11] Bajkin BV, Popovic SL, Selakovic SD (2009). Randomized, Prospective Trial Comparing Bridging Therapy Using Low-Molecular-Weight Heparin With Maintenance of Oral Anticoagulation During Extraction of Teeth. J Oral Maxillofac Surg.

[12] Bacci C, Maglione M, Favero L, Perini L, Di Lenarda R, Berengo M (2010). Management of dental extraction in patients undergoing anticoagulant treatment. Thromb Haemost.

[13] Morimoto Y, Niwa H (2010). On the use of prothrombin complex concentrate in patients with coagulopathy requiring tooth extraction. Oral Med Oral Pathol Oral Radiol Endod.

[14] Pereira CM, Gasparetto PF, Carneiro DS, Corrêa ME, Souza CA (2011). Tooth extraction in patients on oral anticoagulants: prospective study conducted in 108 brazilian patients. ISRN Dent.

[15] Nematullah A, Alabousi A, Blanas N, Douketis JD, Sutherland SE (2009). Dental surgery for patients on anticoagulant therapy with warfarin: a systematic review and meta-analysis. J Can Dent Assoc.

[16] Madrid C, Sanz M (2009). What influence do anticoagulants have on oral implant therapy? A systematic review. Clin Oral Implants Res.

[17] Firriolo FJ, Hupp WS (2012). Beyond warfarin: the new generation of oral anticoagulants and their implications for the management of dental patients. Oral Surg Oral Med Oral Pathol Oral Radiol.

[18] Lam S (2013). Apixaban: A New Factor Xa Inhibitor for Stroke Prevention in Patients with Atrial Fibrillation. Cardiol Rev.

[19] Little JW (2012). New oral anticoagulants: will they replace warfarin?. Oral Surg Oral Med Oral Pathol Oral Radiol.

[20] Eriksson BI, Quinlan DJ, Eikelboom JW (2011). Novel oral factor Xa and thrombin inhibitors in the management of thromboembolism. Annu Rev Med.

[21] Evans IL, Sayers MS, Gibbons AJ, Price G, Snooks H, Sugar AW (2002). Can warfarin be continued during dental extraction? Results of a randomized controlled trial. Br J Oral and Maxillofac Surg.

[22] Méndez C, Cisneros L (2005). Control del sangrado postoperatorio en pacientes anticoagulados empleando colutorios de Acido tranexamico. Odontología Sanmarquina.

[23] Al-Mubarak S, Al-Ali N, Abou-Rass M, Al-Sohail A, Robert A, Al-Zoman K (2007). Evaluation of dental extractions, suturing and INR on postoperative bleeding of patients maintained on oral anticoagulant therapy. Br Dent J.

[24] Spyropoulos AC, Douketis JD (2012). How I treat anticoagulated patients undergoing an elective procedure or surgery. Blood.

[25] Alberts MJ, Eikelboom JW, Hankey GJ (2012). Antithrombotic therapy for stroke prevention in non-valvular atrial fibrillation. Lancet Neurol.

[26] Lillis T, Ziakas A, Koskinas K, Tsirlis A, Giannoglou G (2011). Safety of dental extractions during uninterrupted single or dual antiplatelet treatment. Am J Cardiol.

[27] Madan GA, Madan SG, Madan G, Madan AD (2005). Minor oral surgery without stopping daily low-dose aspirin therapy: a study of 51 patients. J Oral Maxillofac Surg.

[28] Aframian DJ, Lalla RV, Peterson DE (2007). Management of dental patients taking common hemostasis-altering. Oral Surg Oral Med Oral Pathol Oral Radiol Endod.

[29] Krishnan B, Shenoy NA, Alexander M (2008). Exodontia and Antiplatelet therapy. J Oral Maxillofac Surg.

[30] Scharf RE (2009). Management of bleeding in patients using antithrombotic agents: prediction, prevention, protection and problem-oriented intervention. Hamostaseologie.

[31] Pototski M, Amenábar JM (2007). Dental management of patients receiving anticoagulation or antiplatelet treatment. J Oral Sci.

[32] Scully C, Wolff A (2002). Oral surgery in patients on anticoagulant therapy. Oral Surg Oral Med Oral Pathol Oral Radiol endod.

[33] Lockhart PB, Gibson J, Pond SH, Leitch J (2003). Dental management considerations for the patient with an acquired coagulopathy. Part 2: Coagulopathies from drugs. Br Dent J.

[34] Salam S, Yusuf H, Milosevic A (2007). Bleeding after dental extractions in patients taking warfarin. Br J Oral Maxillofac Surg.

